# CATHEDRAL: A Fast and Effective Algorithm to Predict Folds and Domain Boundaries from Multidomain Protein Structures

**DOI:** 10.1371/journal.pcbi.0030232

**Published:** 2007-11-30

**Authors:** Oliver C Redfern, Andrew Harrison, Tim Dallman, Frances M. G Pearl, Christine A Orengo

**Affiliations:** 1 Department of Biochemistry and Molecular Biology, University College London, London, United Kingdom; 2 Department of Mathematical Sciences and Biological Sciences, University of Essex, Wivenhoe Park, Colchester, United Kingdom; European Molecular Biology Laboratory, Germany

## Abstract

We present CATHEDRAL, an iterative protocol for determining the location of previously observed protein folds in novel multidomain protein structures. CATHEDRAL builds on the features of a fast secondary-structure–based method (using graph theory) to locate known folds within a multidomain context and a residue-based, double-dynamic programming algorithm, which is used to align members of the target fold groups against the query protein structure to identify the closest relative and assign domain boundaries. To increase the fidelity of the assignments, a support vector machine is used to provide an optimal scoring scheme. Once a domain is verified, it is excised, and the search protocol is repeated in an iterative fashion until all recognisable domains have been identified. We have performed an initial benchmark of CATHEDRAL against other publicly available structure comparison methods using a consensus dataset of domains derived from the CATH and SCOP domain classifications. CATHEDRAL shows superior performance in fold recognition and alignment accuracy when compared with many equivalent methods. If a novel multidomain structure contains a known fold, CATHEDRAL will locate it in 90% of cases, with <1% false positives. For nearly 80% of assigned domains in a manually validated test set, the boundaries were correctly delineated within a tolerance of ten residues. For the remaining cases, previously classified domains were very remotely related to the query chain so that embellishments to the core of the fold caused significant differences in domain sizes and manual refinement of the boundaries was necessary. To put this performance in context, a well-established sequence method based on hidden Markov models was only able to detect 65% of domains, with 33% of the subsequent boundaries assigned within ten residues. Since, on average, 50% of newly determined protein structures contain more than one domain unit, and typically 90% or more of these domains are already classified in CATH, CATHEDRAL will considerably facilitate the automation of protein structure classification.

## Author Summary

Proteins comprise individual folding units known as domains, with a significant proportion containing two or more (multidomain structures). Each domain is thought to represent a unit of evolution and adopts a specific fold. Detecting domains is often the first step in classifying proteins into evolutionary families for studying the relationship between sequence, structure, and function. Automatically identifying domains from structural data is problematic due to the fact that domains vary substantially in their compactness and geometric separation from one another in the whole protein. We present a novel method, CATHEDRAL, which iteratively identifies each domain by comparing a query structure against a library of manually verified domains in the CATH domain database through computational structure comparison. We find that CATHEDRAL is able to outperform the majority of popular structure comparison methods for finding structural relatives. Furthermore, it is able to accurately identify domain boundaries and outperform other methods of structure-based domain prediction for the majority of proteins. CATHEDRAL is available as a Webserver to provide domain annotations for the community and hence aid in structural and functional characterisation of newly solved protein structures.

## Introduction

Proteins comprise individual folding units known as domains, with a significant proportion containing two or more units (multidomain structures) [1]. Each domain adopts a specific fold, and it is estimated that there are up to several thousand such folds in nature [2–4]. As the domain is thought to be an important evolutionarily conserved unit, several structural classifications, such as SCOP [5] and CATH [6], have sought to cluster them into fold groups and evolutionary families. Although a given pair of structures in these families can diverge below similarities of ≤30% in their sequence, these relatives still maintain a comparable topology or fold in the core of the structure [6,7].

More than 7,000 new proteins structures were deposited in the Protein Data Bank (PDB; http://www.rcsb.org/pdb) [8] in 2005. Furthermore, analysis of version 2.6 (April 2005) of the CATH database shows that nearly 50% of classified structures are multidomain. Although many are close in sequence to previously solved structures, the structural genomics initiatives have concentrated their resources on proteins with low sequence similarity to existing structures. As a consequence, they often require considerable manual analysis to be classified in the CATH domain database. That said, the vast majority of newly solved structures contain previously observed folds, although they are often quite remote homologues. In this situation, structural comparison algorithms can be essential to facilitate the automatic and semiautomatic classification of domains.

**Figure 1:**
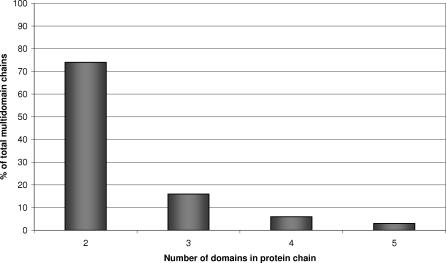
Percentage of Multidomain Chains with a Given Number of Component Domains

The number of larger multidomain structures has been gradually increasing since the formation of the PDB, with improvements in techniques for structure determination. We can expect this trend to continue, as recent analyses of completed genomes have suggested that the proportion of multidomain structures in some organisms, particularly eukaryotes, may be as high as 80% [1]. Figure 1 shows that the majority of multidomain chains comprise two domains, although some structures have been solved with three, four, five, and six domains.

**Figure 2:**
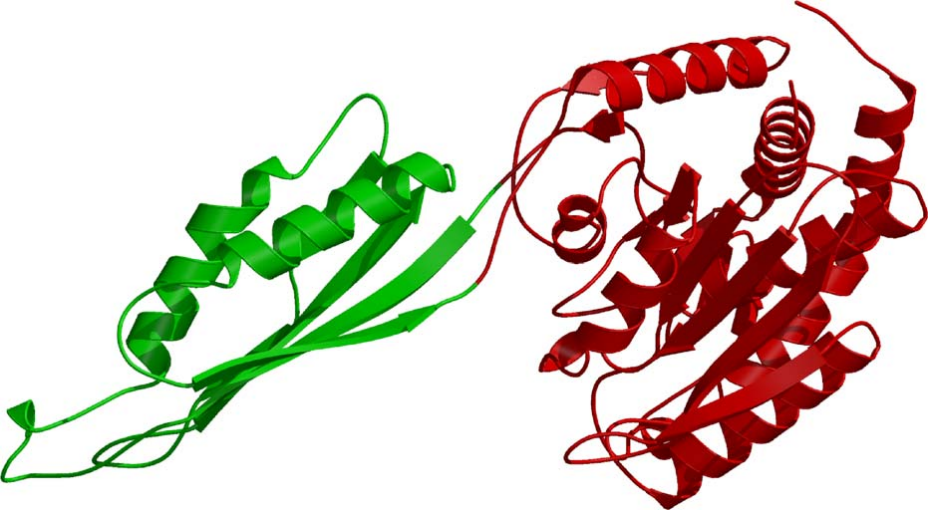
Example of a Multidomain Protein (PDB: 1cg2) Chain Containing a Discontiguous Domain Domain two (blue) is inserted between two segments of domain one (red).

A further complication is that approximately 20% of domains from multidomain proteins in the PDB are discontiguous [9] (Figure 2); that is, the structure of the individual domains is formed from disconnected regions of the polypeptide chain. Both automated and manual approaches to domain boundary recognition have problems in assigning these domains.

Various computational methods have been developed to automatically detect domain boundaries in multidomain structures (see [10]) through a posteriori knowledge of domain folding and interactions. Several approaches assume that a domain makes more internal contacts (intradomain) than external contacts (contact with residues in the remainder of the structure). For example, the DOMAK algorithm of [11] derives a “split value” from the number of contacts measured when a protein is divided into two parts. Optimal values are obtained when the separate parts of the split structure are distinct domains. The protein domain parser (PDP) takes a similar approach and looks to divide multidomain structures so that the number of internal contacts in each putative domain increases. By contrast, the parser for protein unfolding units (PUU) algorithm by [12] uses a harmonic model to describe interdomain dynamics, and is used to define domain units in the FSSP database [13]. A further approach, used by the DETECTIVE algorithm [14], attempts to determine the hydrophobic core at the heart of each domain unit. The original CATH classification protocol [9] used a consensus approach by combining the results from the three independent methods, described above (PUU, DOMAK, and DETECTIVE).

Although each method reports 70%–80% accuracy in benchmarking tests, our experience of updating the CATH database suggest that these methods frequently (∼80%–90% of the time) produce results that are inconsistent with one another. As a consequence, manual validation becomes the only secure way to resolve these conflicting predictions. A recent analysis by Holland et al. [10] showed that all automatic methods run into difficulties when assigning boundaries for certain architectures that do not fit their chosen model of a domain unit—for example, an alpha horseshoe domain, which does not form a compact structure. The authors suggested improvements achieved by a heuristic method that accounts for exceptions to the theoretical domain model. An alternative approach would be to compare a given protein chain against a library of known domain folds.

Although many of the algorithms described above effectively delineate domains for a large percentage of protein chains in the PDB (even those which contain novel folds), they provide no indication as to how similar each predicted domain is to folds already classified within the CATH database. Therefore, it is still necessary to compare the excised domain against the CATH library to classify the fold. Since manual validation of domain boundaries and structure-based database scans are both slow, this has remained one of the major bottlenecks in the CATH classification process.

As discussed above, there are a limited number of folds, and a novel multidomain structure could well comprise those that have already been classified. This concept of recurrence is not new, and has been successfully exploited by other structural classifications. For example, the DALI algorithm is used to detect recurrent folds for classification in the DALI Domain Database [13], while the SCOP database uses manual inspection to locate known folds.

Many methods exist to find recurring domains using pure sequence approaches (e.g., MKDOM [15], SMART [16], and PFam [17]). However, these are designed to identify individual protein families within gene sequences, rather than predict structural domains. Others, such as SnapDragon [18] and Rigden's covariance analysis [19], attempt to infer domain boundaries through prior prediction of tertiary structure. Nagarajan and Yona [20] used a combination of PSI-BLAST multiple alignments, predicted structural features, and neural networks to identify the transition between domains in the sequence (i.e., the boundaries). The authors were able to correctly predict the domain architecture for 35% of multidomain proteins when compared with SCOP.

Recent analyses of structures solved by the structural genomics initiatives—which are frequently targeted because they have no clear sequence similarity to existing structures and may adopt novel folds—show that approximately 90% are similar to those already observed in the PDB through sequence or structure comparison [21,22]. Therefore, exploiting the concept of domain recurrence to detect known folds in newly determined multidomain structures is a sensible strategy to classify the majority of new structures. Moreover, several fast and powerful algorithms for structure comparison now exist that could be used to perform this task. Some of these compare secondary structures between proteins [23–25], while others operate at the residue level (DALI [26], SSAP [27], COMPARER [28], STRUCTAL [29], and CE [30]).

The performance of an automatic structural alignment method should be measured both on its ability to generate biologically meaningful alignments and its capacity to accurately detect fold similarities and homologous protein structures. As Kolodny et al. [29] highlight, not all structural comparison methods are as good at scoring their alignments as they are at producing them. A root-mean–squared deviation (RMSD) value, or any linear transformation of this, often remains dependent on the number of aligned residues. Some algorithms (e.g., CE [30]) are optimised to find highly conserved regions between two protein structures with a low RMSD. This can be useful in detecting similarities within extremely diverse superfamilies and fold groups. However, this approach does not necessarily give a globally optimal alignment, and can assign high significance to matching small structural motifs that may not be in equivalent positions in the two structures being compared. Hence, for the purpose of domain boundary recognition, it is also vital to consider the number of aligned residues as a proportion of those residues in the larger of the two structures as well as the RMSD of superposed residues.

This paper reports the development of the CATHEDRAL algorithm, a novel domain identifier that exploits the fold-recurrence philosophy. CATHEDRAL is an acronym for CATH's existing-domain recognition algorithm. It compares a novel multidomain protein structure against a library of previously classified folds in the CATH database [6] by modifying and combining features from two established structural similarity algorithms. A secondary-structure–matching algorithm, GT (using graph theory) [25], which is very fast and reasonably accurate, is combined with a residue-based method that uses double-dynamic programming (DDP) [27], and is therefore slower but very accurate. By combining these approaches, a 100-fold to 1,000-fold increase in speed is achieved, depending on the size of the query structure, at no cost to the quality of the domain alignments. This enables regular scans of newly determined protein structures and rapid classification of their constituent domains into the CATH database.

To investigate the efficacy of CATHEDRAL in producing quality alignments, it has also been benchmarked against other publicly available structure comparison algorithms at the single-domain level. By aligning domains in a consensus SCOP/CATH dataset, CATHEDRAL was found to give comparable and, in many cases, superior performance for fold recognition. In addition, when assessing the fidelity of the structural alignments in comparison to hand-curated structural alignments with respect to BAliBASE [31], it consistently performed better than other approaches by aligning more residues correctly.

## Results

### Evaluating the Performance of the GT and DDP Algorithms for Fold Recognition at the Domain Level Using a Consensus CATH–SCOP Dataset

The rationale behind CATHEDRAL was to use a fast secondary structure–based graph theory (GT) algorithm to discover putative fold matches for a given protein domain/chain structure, which could subsequently be more accurately aligned using a residue-based method exploiting DDP. To evaluate the performance of GT and DDP for fold recognition and accurate alignment, we first created a dataset of single CATH–SCOP domains and compared this approach with other publicly available methods before optimising the algorithm for discovering domain folds in multidomain chains. This benchmarking was also performed on a larger dataset of domains in the nonredundant CATH library (version 2.6) and produced almost identical results.

#### Comparing DDP and GT.

The coverage and precision of the GT and DDP algorithms was compared by scanning the benchmark CATH–SCOP dataset (see Methods) against a library of CATH domains and ranking the results by the GT E-value and the DDP structural alignment score (SAS; see Methods for descriptions of scoring schemes). It can be seen from Table 1 that the DDP algorithm finds the correct fold as the top hit nearly 95% of the time compared with nearly 84% for the GT method. Nevertheless, the correct fold is within the top 10 hits for GT nearly 98% of the time*.* Given that the DDP algorithm is 100–1,000 times slower than GT, depending on the size of the structures being compared, taking the top ten folds identified by GT forward for more accurate scoring by DDP maintains coverage yet vastly reduces the number of DDP comparisons undertaken. We subsequently refer to the combined use of GT and DDP as CATHEDRAL.

**Table 1:**
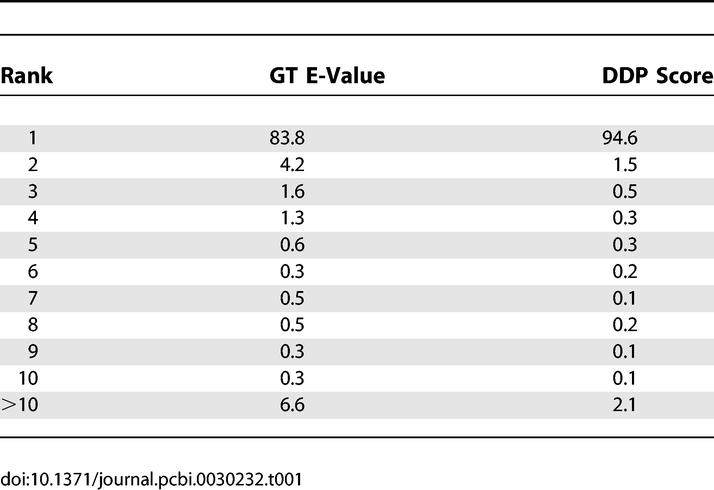
Percentage of Correct Folds Identified by GT and DDP

### Comparing the Performance of the Component Algorithms in CATHEDRAL (GT, DDP) with Other Established Structure Comparison Algorithms

#### Fold recognition.

To benchmark CATHEDRAL, we compared its performance against several other publicly available methods (DALI, STRUCTAL, LSQMAN, and CE) using the same CATH–SCOP dataset (see Methods). Kolodny et al. [29] have also recently benchmarked these methods using various scoring schemes, and they found that a normalised RMSD score (SAS; see Methods), performed best for recognising domains with the same fold.

**Figure 3:**
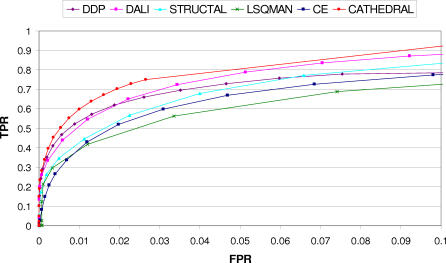
ROC (True Positive Rate Versus False Positive Rate) Curve Plotted for Different Structural Comparison Methods Based on the SAS, Where a Positive Match Represents a True CATH–SCOP Fold Match TPR, true positive rate; FPR, false positive rate.

It can be seen from Figure 3 that CATHEDRAL returns the highest proportion of true positives for a 5% error rate, followed by DALI, DDP, and STRUCTAL. It is interesting to note that CATHEDRAL actually outperforms DDP. This is probably because it excludes potentially low-scoring comparisons at an early stage in the algorithm, thus reducing the number of false positives, while the other methods return scores for all comparisons.

As well as the ability of the SAS to correctly discriminate between true and false fold matches across a range of score cutoffs, it is important to identify the closest relative within a given fold group to obtain the best alignment. Therefore, the correct fold should rank highly in the list of matches.

**Figure 4:**
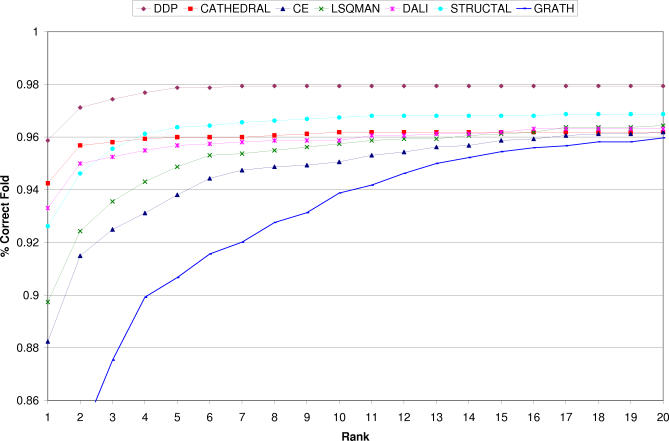
Graph of the Percentage of Correct Folds Matched Against the Ranked Native Score for the CATH–SCOP Dataset

It can be seen from Figure 4 that CATHEDRAL assigns the correct fold as its top hit more than 94% of the time compared with the best method DDP, at 96%. When the percentage of correct fold matches with the top ten matches are considered, the CATHEDRAL performance rises to 96% and DDP to 98%, with STRUCTAL also slightly outperforming CATHEDRAL. This small discrepancy is mainly for small domains with few secondary structures (less than four), which cannot be compared using GT (see Methods). The performance could relatively quickly be recovered by comparing domains with less than three secondary structures against a library of small domains using just DDP.

#### Assessment of alignment quality.

Alignment accuracy was initially assessed using a similar strategy to that devised by Kolodney et al. [29]. The authors measured the proportion of correct fold matches producing a structural similarity score below a given threshold. In assessing DDP in this way, we have also chosen to use the SAS they propose and a new score, SiMax (see Methods). The latter is a modified version of the SiMin score used by Kolodny et al. SiMax normalises by the larger of the two domains being compared rather than the smallest as with SiMin. This allows it to better determine whether methods are good at recognising the best “global” as opposed to “local” fold similarity.

**Figure 5:**
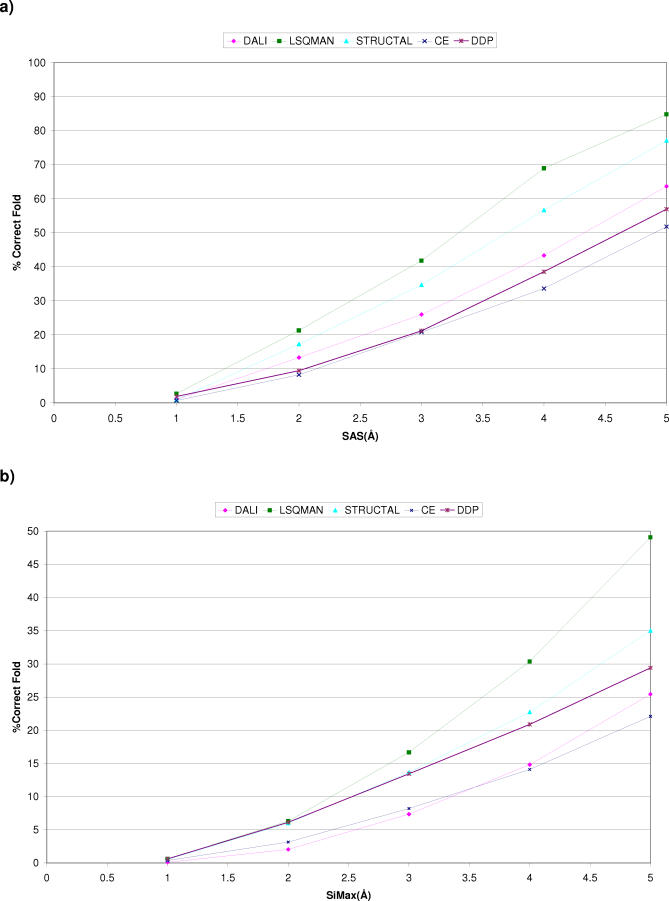
Comparison of Alignment Quality of Domains Adopting the Same CATH Fold Using Two Geometric Scoring Schemes (A) Percentage of correct fold pairs for a given SAS threshold. (B) Percentage of correct fold pairs for a given SiMax threshold.

Figure 5A and 5B show alignment quality as measured by the SAS and SiMax for domains in the same fold. The best performing method using both scores is LSQMAN, although it is known to align many fewer residues than the other methods. Given that it is one of the worst performers in the receiver operating characteristic (ROC) curve analysis, this suggests that using an RMSD-based score alone as an assessment of alignment quality does not necessarily correlate with a globally optimal alignment. Structural variation within fold groups and superfamilies can result in high RMSD values for otherwise good alignments with algorithms that seek to maximise the number of equivalent residues. For example, LSQMAN looks to align fragments with low RMSDs, and consequently can restrict the number of aligned residues. Although this can be useful for detecting domains sharing common motifs, it will often not identify all biologically equivalent positions—information that is essential for domain boundary recognition.

In some domain architectures (e.g., three-layer αβ sandwiches), it is clear that large structural motifs (e.g., βαβα) can overlap between domains in different folds, yet these do not always coincide with equivalent secondary structures. Furthermore, any similarity score based on RMSD will be dependent on the number of superposed residues, and hence aligning more residues in variable parts of two structures can give a disproportionately high score, even if the alignment is actually more biologically relevant. For the purposes of applications such as domain boundary recognition, it is clearly important to identify an alignment between two domains that maximises the number of equivalent residues. Consequently, for a given pair of fold matches, we looked at the average number of residues aligned by each method. Table 2 shows this calculation relative to DDP, as DDP aligns more residues than all other methods. What is interesting is that there appears to be a correlation between this average value and the SAS ROC curves shown in Figure 3. More specifically, DALI, STRUCTAL, and DDP align the most residues and also perform best under the ROC curve analysis.

**Table 2:**
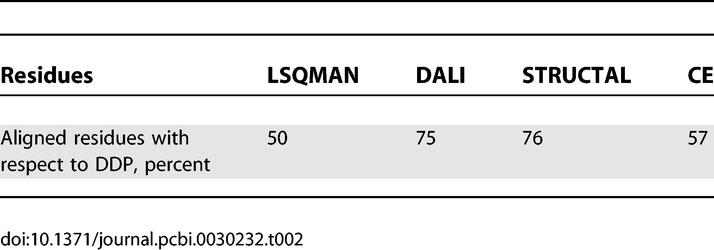
Residues Aligned by Each Method Relative to DDP for All Genuine Fold Matches

**Figure 6:**
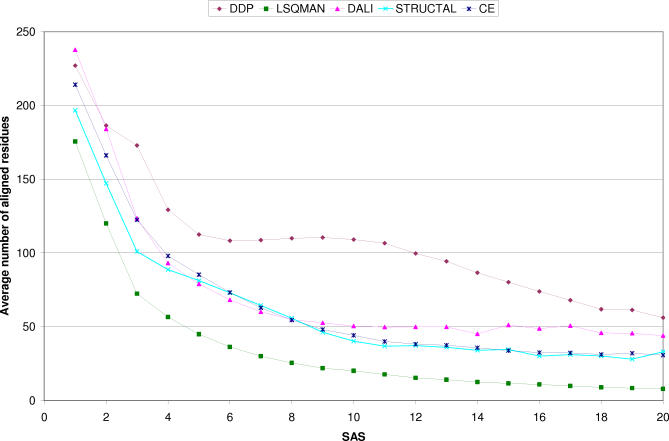
Average Number of Aligned Residues per SAS

We also examined the size of the domain fragments aligned with a given SAS. Figure 6 shows that although LSQMAN and STRUCTAL are returning a higher proportion of scores below a given SAS/SiMax threshold (Figure 5), they recognise and align fewer residues. This may be valuable for finding the most conserved structural motif between two domains; however, it is less useful for assigning domain boundaries. Taken together, Figure 5 and Figure 6 suggest that DDP is a suitable method to use for domain boundary recognition, as it aligns large fragments while ensuring these can be superimposed with reasonable scores.

#### Comparison with manually curated alignments.

**Figure 7:**
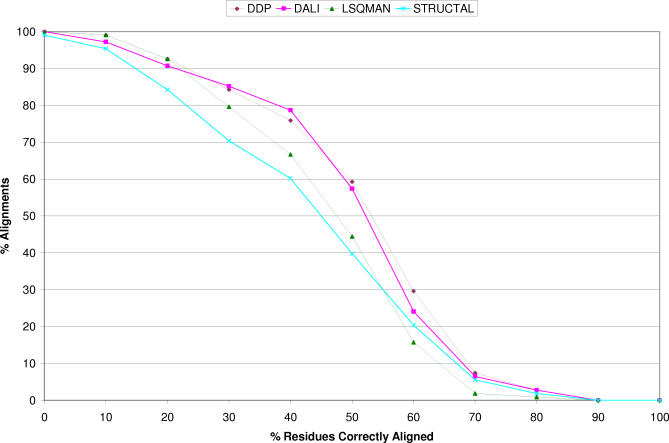
Graph Showing How the Alignments of Each Method Compared with Manually Validated BAliBASE Alignments The higher the curve (or the curve with the greatest area underneath) represents the method that most agrees with the manually curated BAliBASE alignments.

A better way to assess the biological significance of automatic structure alignments is to compare them to a manually validated dataset. To this end, we compared all methods against curated alignments from the BAliBASE (see Methods). Figure 7 shows that DALI and DDP produce alignments closer to the BaliBASE alignments, with nearly 60% of DALI and DDP alignments having at least 50% residues correctly aligned, compared with 45% for LSQMAN and 40% for STRUCTAL. The data from Figures 5–7 suggest that LSQMAN and STRUCTAL may be optimising alignments to reduce the RMSD at the expense of finding the most biologically equivalent residues.

In summary, our benchmarking results suggest that GT and DDP are complementary approaches that may be combined to produce a fast yet reliable domain boundary recognition algorithm. The performance of CATHEDRAL for this purpose is described in the next section.

### Performance of CATHEDRAL for Domain Recognition in Multidomain Protein Chains

#### Overview of the protocol adopted by CATHEDRAL.

The preceding sections dealt with the use of the CATHEDRAL protocol at the *domain* level to identify fold relatives in a domain library. In this section, we develop CATHEDRAL to scan a novel *multidomain* structure against a library of representative single-domain folds in order to identify constituent domains, thereby locating domain boundaries. A GT approach is initially used to find putative folds in CATH within the protein structure. Representatives from each superfamily in each of the high-scoring fold groups are then aligned against the putative domain region in the chain using a more accurate, residue-based DDP algorithm. DDP was modified to allow the common secondary structures determined by GT to guide a residue alignment by DDP, which reduces the search space and hence results in a notable speed increase. The significance of the subsequent matches is assessed using a support vector machine (SVM), which accounts for the percentage of overlapping residues, geometric similarity, and number of aligned residues. Once a domain has been assigned, it is excised from the chain, and the remaining regions are iteratively recompared using the same protocol until all known domains have been found. An optimal balance between coverage and speed was achieved by only realigning domains in the top ten folds identified by GT using DDP.

#### Optimising alignment scoring using an SVM.

As discussed above, it is essential to consider both the RMSD of the structural alignment of two domains, as well as the percentage of aligned residues in the largest protein. This problem becomes more complex when detecting individual domains in a multidomain chain, as the constituent domains may be unknown. When comparing a chain with a domain library, the best RMSD can sometimes be obtained by small domains with similar secondary structure motifs but different folds, which might inhibit the detection of truly equivalent domains.

The highest-scoring match recognised by CATHEDRAL is used to determine the boundaries of the domain. Residues associated with that domain are then excised from the multidomain chain before rescanning the chain against the fold library. Therefore, it is important that this top match is frequently the best global match that can be obtained. As well as using information on the number of residues that superpose well (provided by the SAS), it is also important to consider the proportion of residues equivalent between the two domains, domain sizes, and protein classes. All these features have been found to be important in the manual curation process used to classify domains in CATH. We used an SVM to explore whether some optimal combination of these features resulted in a measure that was more discriminating than simply using a single structural similarity score.

**Figure 8:**
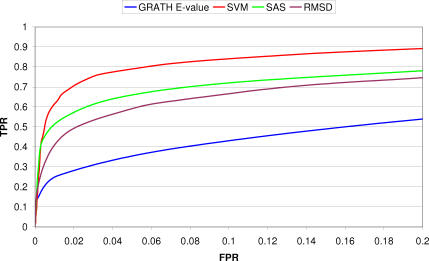
Comparison of GT and DDP Scores with SVM Score for Assigning Domains to Multidomain Chains

From Figure 8, it can be seen that using an SVM to combine the other scoring schemes into a single value outperforms the individual methods at low error rates. The SVM ROC curve benchmark shown is an average of the performance shown using 5-fold cross-validation to account for any overfitting of the dataset. It is also interesting to note that the GT E-value actually outperforms the SAS at error rates higher than 2%.

### Optimising a Score Threshold for Accurately Identifying Domains in Multidomain Chains

**Figure 9:**
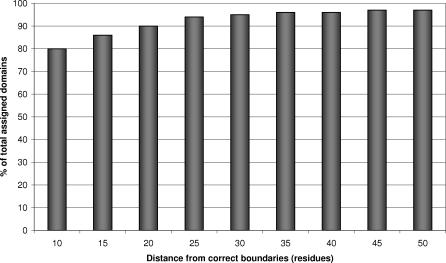
Percentage of Domain Assigned (Blue) and Percentage of Domain Boundaries within Ten Residues of Verified Boundaries (Pink) at a Range of SVM Score Cutoffs

**Figure 10:**
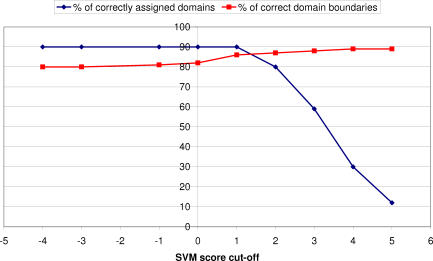
Domain Coverage Versus Quality of Domain Boundaries

Once CATHEDRAL has identified putative domain matches for a query multidomain structure, all domain hits to the chain are ranked by the SVM score, and domain boundaries are assigned using the protocol described in Methods. CATHEDRAL was able to assign 90% of domains in the query dataset to the correct fold group, with 80% of these within ten residues of the actual boundary (Figure 9). Although our dataset only contained multidomain chains in which all component domains were represented in the CATH library, this is not always the case in classifying novel structures. Indeed, assigning erroneous folds to chains could adversely affect the quality of the domain boundaries. Figure 10 shows a plot of coverage according to the percentage of accurate boundaries (i.e., within 10 residues). It can be seen that once the SVM score cutoff is increased above 2, the coverage drops dramatically. However, the accuracy of the domain boundaries does not increase significantly, suggesting that this is an appropriate threshold for CATHEDRAL. Figure 9 shows the coverage of all chains in the dataset with respect to the accuracy of their predicted domain boundaries.

CATHEDRAL was developed as a method to be applied unilaterally to all protein chains to be classified into the CATH database. As it is not known a priori whether a given chain contains more than one domain, it is important that the algorithm does not split whole-chain domains unnecessarily. To analyse whether this would pose a problem, the iterative version of CATHEDRAL was also applied to the single-domain CATH–SCOP dataset. In less than 4% of cases, CATHEDRAL predicted that these structures contained more than one domain.

### Increasing the Speed of Domain Boundary Assignments by CATHEDRAL

The major speed increase in CATHEDRAL is due to the fact that GT preselects representatives for DDP to align to the query chain. By default, it takes all relatives (nonredundant at 35% sequence identity level) in each of the top ten top-scoring fold groups identified by GT, even if this results in thousands of comparisons, as occurs in large fold groups such as the Rossmann and TIM barrel folds. This can produce very long running times for some query chains. Nevertheless, it is important to find the closest structural relatives for each assignment to reduce the number of insertions and deletions and therefore increase the accuracy of the domain boundary.

We explored whether only a limited number of relatives from each fold could be taken without compromising the fidelity of the domains boundaries. However, given that GT does not accurately discriminate between homologues and domains with the same fold, it was decided to take at least one relative from each superfamily in the target fold group and explore the effect of varying this number.

**Figure 11:**
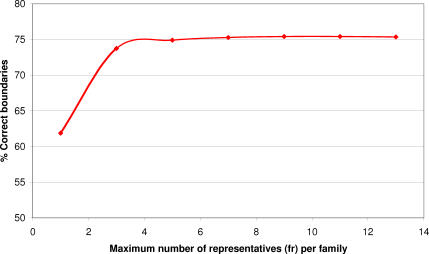
Percentage of Domains with Correct Domain Boundaries (within Ten Residues) When Varying the Number of Representatives Taken from Each Superfamily in the Targeted Fold Groups

CATHEDRAL was run as described above (by targeting the top ten fold groups at each iteration), but the number of nonredundant representatives (*fr*) taken from each superfamily to be aligned by DDP was varied. Figure 11 shows a plot of the number of correctly assigned domain boundaries (within ten residues of manually validated boundary) at each of these levels. It appears that taking any more than seven representatives from each superfamily does not increase the number of good assignments, and hence appeared to be an appropriate level to set the *fr* parameter.

### Relationship between the Accuracy of Assigned Domain Boundaries and Evolutionary Distance from the Matched Domain

**Figure 12:**
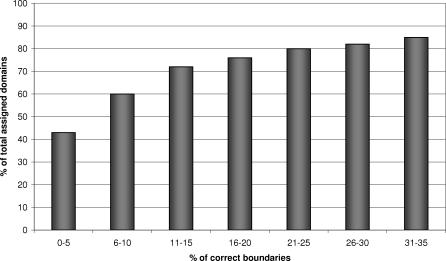
Graph of the Percentage of Correct (within Ten Residues) Domain Boundaries against the Sequence Identity between the Assigned Region and the Matched Domain

Figure 12 shows the relationship between the accuracy of the domain boundary and the sequence identity between the assigned domain region and best structural match used to assign the boundary. When sequence identity exceeds 10%, there is an increase in the number of correct domain boundaries. It could be expected that the closer the relative from which the assignment is made, the greater chance of it being correct. However, it is encouraging to note that 60% of assignments with sequence identities between 5% and 10% show very little deviation from the manually verified boundaries.

**Figure 13:**
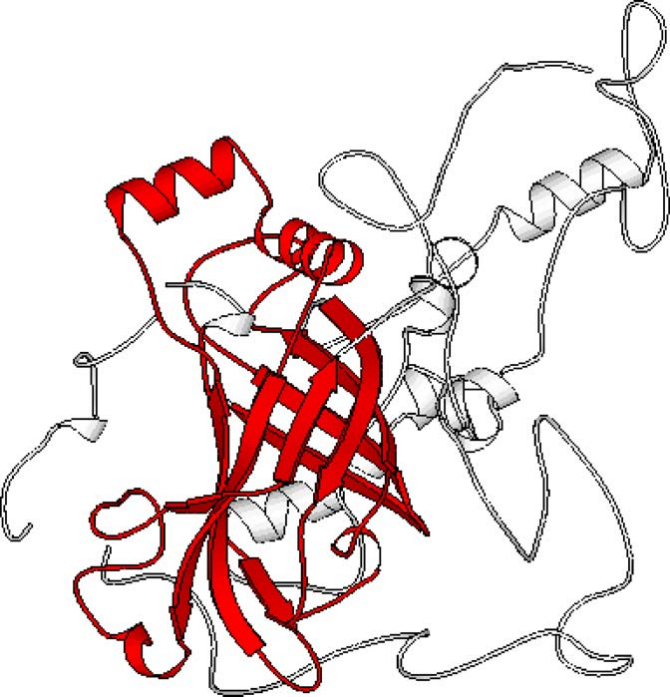
Superposition of the Catalase HPII (PDB 1iph; First Domain of Chain A) as It Is Classified in the CATH Database and Its Match to Bovine Beta-Lactoglobulin, Coloured Red, (PDB 1beb; Chain A), the Closest Relative Identified by CATHEDRAL

Structural embellishments of the core of a fold are responsible for the majority of examples where there is a disagreement between a manually assigned boundary and those predicted by CATHEDRAL*.* Figure 13 illustrates this problem by showing a domain assignment for a catalase HPII [32] (PDB code 1iph) domain, through similarity to its closest match in the CATH library [33] (PDB code 1beb). The matched domain is much smaller than the query, and hence CATHEDRAL is only effective at aligning the core of the fold (shown in red). A number of large insertions in the catalase domain cannot be assigned purely by structural comparison, and these sites are therefore not included within the domain, causing a substantial discrepancy from the correct boundary assignment.

Recent analyses of CATH superfamilies has revealed that in 40% of well-populated superfamilies (nine or more diverse relatives at <35% sequence identity), there is 2-fold or more variation in the sizes of the domains (as measured by the numbers of secondary structures in the domain) [7]. Therefore, in these superfamilies, it may be difficult to obtain accurate boundaries until a close structural relative is deposited in the PDB.

### Comparison of CATHEDRAL Performance to Domain Boundary Assignment by Other Domain Prediction Methods

To place the performance of CATHEDRAL in context, we compared its ability to assign domains boundaries with two other methods: hidden Markov models (HMMs) and domain predictions from structure (PDP).

Our dataset of protein chains was scanned against HMMs built from each structure in the CATH library using the HMMer suite of programs [34]. Domain boundaries were then assigned to the query chains in the same way as CATHEDRAL, but using the HMM E-value instead of the CATHEDRAL SVM score to rank hits. We found that the HMM method was only able to discover 65% of domain folds within the dataset chains. One of the main reasons for this low coverage was that 11% of the chains were not annotated with any domains using an E-value threshold of 0.001. Of the domain boundaries assigned, only 33% were within ten residues, compared with 80% for CATHEDRAL. It is possible that the number of assigned domains could have been increased by using a less conservative E-value threshold. However, this is unlikely to improve the overall quality of the domain boundaries, given the low quality of those that were assigned by the HMM alignments. The domain recognition performance is on a par with the method of Nagarajan and Yona [20], who predicted the correct domain architecture of 35% of a dataset of multidomain PDB chains. However, by incorporating structural information they were able to increase the percentage of boundaries within ten residues to 63%.

CATHEDRAL finds domain boundaries for a query chain by using structural alignment to known folds in CATH. To compare our approach with other methods that do not exploit the concept of fold recurrence, but instead are based on ab initio analysis of structural properties such as residue contacts, we applied the PDP method to our multidomain chain dataset. PDP was able to predict correct domain boundaries (within ten residues) for 67% of the chains in the dataset. Although this is lower than CATHEDRAL, it is substantially higher than the 33% achieved by HMM methods. Furthermore, the performance of PDP is still impressive given the problem of distinguishing domain units in a chain based purely on structural properties such as internal contacts and hydrophobicity.

### CATHEDRAL Webserver

More than 50 structural comparison algorithms have been published in the literature in the last 30 years, the vast majority of which are not in regular use by the bioinformatics or structural biology communities. Those which have gained popularity tend to have a Web-based interface for users to submit their own structures or structures from the PDB. CATHEDRAL has been implemented as a crucial part of the CATH classification protocol, and a new Webserver was created to provide users to investigate domain assignments and homologue recognition with their protein structure of choice (http://cathwww.biochem.ucl.ac.uk/cgi-bin/cath/CathedralServer.pl).

## Discussion

We have developed a protocol for domain boundary assignment in multidomain proteins (CATHEDRAL) that exploits the recurrence of folds in different multidomain contexts. This was devised because a high proportion (currently >90% [21]) of domains in newly determined structures contain folds that have been previously classified in CATH.

CATHEDRAL first scans a query structure against a library of folds from the CATH databases. The algorithm first uses GT to perform a secondary structure–based comparison and identify putative domain fold matches in the query structure. A representative sample of nonredundant superfamily relatives from the top ten folds are then recompared to try to obtain a better alignment and refine the domain boundaries. This latter step exploits a DDP algorithm that has been guided by information on equivalent secondary structures identified by the GT match.

CATHEDRAL combines the power of two established structural comparison algorithms in order to develop a fast and accurate protocol for homologue recognition and domain assignment. CATHEDRAL misses ∼10% of the domains in the target dataset. Of these, ∼30% are too small (fewer than three secondary structures) and so are ignored by the CATHEDRAL protocol. Manual inspection revealed that a further ∼20% are distorted or irregular structures giving poorly defined graphs. The remaining ∼50% are missed because they do not pass the score similarity cutoff, as the relatives are too distant and related structural motifs in neighbouring fold groups are better matched.

The CATH classification of protein folds gives a discrete description of fold space. However, there are difficulties in identifying distinct folds in some populated regions of fold space where the structural universe could be more reasonably represented as a continuum [6]. In many cases, as the size of the protein increases, the repertoire of folds appears to consist of extensions to existing motifs. It has been shown by Koppensteiner et al. [35] that it is possible to “walk” from one α/β sandwich fold to another, through the extension of α/β motifs. Furthermore, certain motifs, described as “attractors,” occur as the core of a protein's structure more frequently than others [36]. Recent analyses of the overlaps between fold groups has shown that for some protein architectures (αβ sandwiches and mainly β sandwiches), extensive overlaps between fold groups are observed due to large common structural motifs [37].

For nearly 80% of the test set, all domain boundaries within the multidomain were correctly assigned within ten residues. This is a considerable improvement over a previous consensus protocol (DBS; [9]) described above, for which on average only 10%–20% of domains could be identified as having reliable boundary assignments from agreement between three independent methods. Furthermore, as known folds are recognised by CATHEDRAL, individual domains can be simultaneously classified in the CATH database, without the need for further structure comparison as in previous classification protocols. The method is currently being extended to assign a confidence level or *p*-value to the boundary and fold assignments predicted by CATHEDRAL. Furthermore, at present, CATHEDRAL assigns domains to a query chain in an iterative fashion. It could be conceived that a better prediction of boundaries and fold assignments could be attained by considering a number of different classifications. The best of these could be identified as the prediction with the highest confidence value.

Since CATH aims to maintain high quality domain boundary assignments [38], results returned by the CATHEDRAL algorithm will continue to be manually assessed. However, the high accuracy of the approach will considerably facilitate this process. Since the proportion of domain folds classified within CATH is likely to continue to increase significantly in the next decade due to the progress of the structural genomics initiatives, the CATHEDRAL algorithm will considerably increase the speed of classification of new multidomain structures and their constituent folds within CATH.

## Methods

### Generation of a dataset for benchmarking structure alignments of single domains.

CATHEDRAL and DDP (a modified version of the SSAP algorithm [27]) were benchmarked against other publicly available structural comparison methods, STRUCTAL [29], DALI [26], LSQMAN, and CE [30] (see Text S1 for description of methods). An all-against-all structural comparison was performed on the 6,003 unique CATH domains (<35% sequence identity to each other) from 907 fold groups for each of the different structural comparison methods, giving more than 18 million individual comparisons. To minimise any bias in the CATH dataset, a dataset that was a subset of CATH version 2.6.0 and SCOP verson 1.65 was also constructed. Each of 6,003 CATH (SRep) domains was checked to see if it had an equivalent SCOP domain containing at least 80% of the same residues. All domains satisfying this criterion were mapped to their CATH and SCOP superfamilies. These superfamilies were then compared, and only those sharing 80% of the same members were identified. This restricted the CATH–SCOP dataset to 1,779 SReps encompassing 406 folds.

### Overview of the publicly available methods used to assess the relative performance of CATHEDRAL and DDP for domain–domain structure alignment.

There are several publicly available methods that have been endorsed by widespread community use and/or validation by comparative benchmarking against established methods. We selected a range of methods, many of which had been previously benchmarked by Kolodny et al. [29] for their performance in fold recognition and alignment accuracy.

*Protocol used to compare the performance of CATHEDRAL and full DDP in fold recognition and alignment accuracy with other established methods—fold recognition.* Structure alignment methods were compared using ROC curves. These plot true positive rate (sensitivity) against the false positive rate (1 − specificity) for different similarity scores returned by the individual methods. A binary classifier was defined by the CATH hierarchy whereby a positive match is one in which both domains share the same fold classification. The matches for each method were ordered by the structural similarity score of their alignment, and the number of true positives and false negatives were calculated at varying thresholds.

*Protocol used to compare the performance of CATHEDRAL and full DDP in fold recognition and alignment accuracy with other established methods—alignment accuracy.*

Kolodny and coworkers tested several measures for assessing the accuracy of structural alignments [29]. They identified redundant measures, and alignment accuracy was subsequently compared using the two geometric measures shown in Equations 1 and 2 below: SAS, and SiMin where *N_mat_* represents the number of aligned residues and *L_1_*/*L_2_* represents the length of the respective domains. The different measures attempt to balance the different properties that describe a “good” alignment, weighting the RMSD against the length of the alignment as a fraction of the length of the proteins aligned.

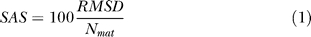


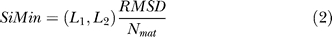



As CATHEDRAL is exploiting fold recognition to obtain reliable domain boundary assignment, we developed a further measure that scores the global alignment accuracy. As opposed to SiMin, which gives a good score for a small fold appearing as a conserved motif within a much larger fold, SiMax (Equation 3 below) takes account of the proportion of residues aligned in the larger domain structure to determine whether a significant fold match has been achieved.

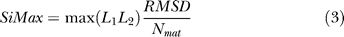



All the measurements are in angstroms, and the percentage of alignments within a particular distance in angstroms were calculated for each measure (SAS, SiMax, and SiMin).

In addition to these geometric measures, alignment accuracy was also assessed by comparison against a set of manually curated alignments. BAliBASE is a database of manually refined multiple structure alignments specifically designed for the evaluation and comparison of multiple sequence alignment programs. The alignments in BAliBASE are selected from the FSSP [36] or HOMSTRAD [39] structural databases, or from manually constructed structural alignments taken from the literature. When sufficient structures are not available, additional sequences are included from the HSSP database [40]. The VAST Webserver [23] is used to confirm that the sequences in each alignment are structural neighbours and can be structurally superimposed. Functional sites are identified using the PDBsum database [41], and the alignments are manually adjusted to ensure that conserved residues and secondary structure elements are correctly aligned.

A total of 14 BAliBASE multiple alignments were selected, comprising 108 pairwise structural comparisons. All the alignments represented single-protein domain chains that shared less than 25% sequence identity, making alignment nontrivial. All three major protein classes were represented, and the quality of the alignments generated by the different structure comparison methods are measured by the score, *f_m_*, which quantifies the number of amino acids correctly aligned in the structural alignment divided by the total number of aligned residues in the BAliBASE alignment. CE was not included in this analysis, as it only identifies the largest continuous motif.

### Generation of a dataset used in benchmarking CATHEDRAL for assigning folds to multidomain protein chains.

CATHEDRAL was benchmarked to calculate its ability to delineate domains within multidomain proteins, as well as correctly recognising the fold of the constituent domains. A set of representatives from 1,071 multidomain S35 sequence families (clustered by single linkage at 35% sequence identity) was selected. From this set, those chains containing domains from folds with less than two S35 sequence families were removed. The remaining set contained 964 chains comprising 1,593 domains. These originated from 245 distinct fold groups and 462 superfamilies.

### Generation of a CATH library of representative folds for CATHEDRAL.

To identify domain boundaries in a novel multidomain structure, CATHEDRAL scans the query structure against a library of folds classified in the CATH database (see Text S1 for description of CATH hierarchy) that are derived from contiguous domain representatives from each sequence family (in which relatives have at least 35% sequence identity) in version 2.6 of the CATH database. This comprised 4,707 domains, covering 907 folds. A secondary structure graph of each domain was generated as described in Harrison et al. [25].

### Overview of the CATHEDRAL algorithm.

**Figure 14:**
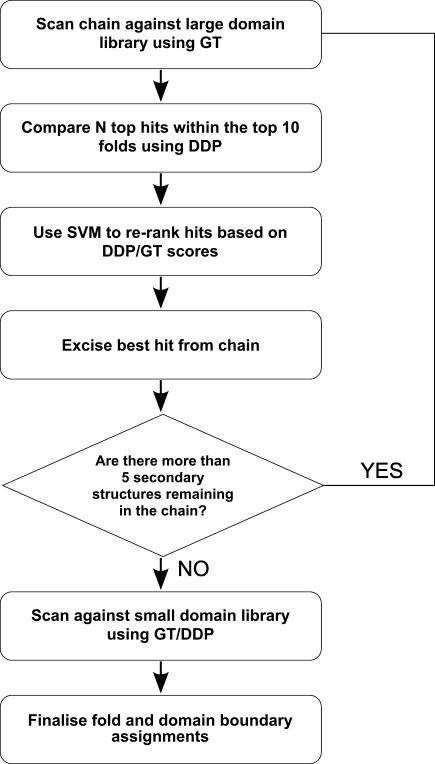
Flowchart of CATHEDRAL Algorithm for Assigning Folds and Domain Boundaries to Protein Chains

*Iterative protocol used by CATHEDRAL.* CATHEDRAL uses an iterative protocol illustrated in Figure 14. As described above, novel multidomain proteins are first scanned against a library of domain folds from CATH using the secondary structure GT algorithm. All folds containing hits in the top ten ranked fold hits are then selected for further analysis. To improve the alignment of the matched regions and thereby identify the closest structural neighbour, *fr* representatives from each superfamily in the selected folds are compared against the matched region using the DDP algorithm.

As matches to small domains (fewer than five secondary structures) can produce insignificant E-values (see [25]) when compared to large chains, these were isolated from the original CATH library and scanned only after all large domains had been assigned by CATHEDRAL.

A variety of different scoring schemes were assessed for their ability to recognise true matches, together with a combination of several measures using an SVM (see below). If the score suggests that the match is valid, the region is accepted as a putative domain and the alignment used to indicate the residues that can be excluded from the multidomain structure (and score matrix) in future searches. A new graph is constructed from the remaining secondary structures, and the GT and subsequent DDP search is repeated to identify another putative domain. CATHEDRAL continues for up to ten iterations or until there are fewer than three secondary structures left to be assigned.

*Identification of corresponding secondary structures in the multidomain protein and a single domain structure using GT.* GT was first introduced for protein structure comparison by Artymiuk and coworkers [42]. CATHEDRAL uses a new implementation of this approach [25] (see Text S1) that includes further structural features (e.g., chirality) to obtain a better resolution between related and unrelated folds. A robust statistical framework was also derived to calculate expectation values (E-values) that can be used to assess the significance of each comparison (see [25] for a detailed description of the GT algorithm used in CATHEDRAL).

*Generating a residue alignment of the fold match using DDP.* Once a putative domain within the multidomain structure has been matched to a fold in the CATH database, an accurate alignment between this domain and the target structure can be obtained using a residue-based method that exploits DDP.

CATHEDRAL uses the global alignment version of the DDP algorithm, described in Taylor and Orengo [27]. This choice followed assessment of the performance obtained using the global and local alignment versions [43]. The global alignment version is better able to handle proteins with discontiguous domains, as the alignment produced by the local version in such cases was found to match only one of the fragments of the discontinuous domain. The break in the discontiguous domain appears to the alignment program as a large gap, and the local alignment score within the gapped region rapidly falls to zero, thus terminating the alignment incorrectly.

*Using secondary structure matches from the GT filter to guide residue alignment by DDP.* The full DDP algorithm is computationally expensive because it makes an exhaustive search of all possible pathways through the residue and summary level matrices, although this search can be constrained by imposing a window on the score matrix [27]. Fortunately, it is not necessary to compare all the equivalent positions between two related proteins to obtain an accurate residue alignment. Therefore, the clique information identifying matching secondary structures can be used to exclude large regions of the score matrix by populating a binary matrix, which dictates which residues to compare. First, residues in equivalent secondary structures must pair with one another. As equivalent strands and helices can vary in length (e.g., a helix with 11 residues could be aligned to one with eight residues), it must be an all-versus-all pairing (represented by a square of “1” values in the matrix). Similarly, residues on the end of aligned secondary structures could potentially be paired with residues in the loop regions, so the boundary is extended by 10 residues on either side.

Second, although the alignment for residues outside the clique is unknown, it is possible to exclude certain pairings. The clique effectively orientates the alignment and dictates that if helix 1 in protein A is equivalent to helix 2 in protein B, it cannot simultaneously be equivalent to helix 3 in protein B. Moreover, it gives the overall direction of the alignment and allows the regions between the clique secondary structures to be linked together.

Finally, the alignment of embellishments of the core clique secondary structures at the start and ends of the domains is unspecified. However, it is known that these cannot be aligned to any of the core residue pairs. Hence, the starts and ends of the domains are paired up for DDP to decide where the equivalences lie. As outlined in the DDP description in Text S1, residue pairs possessing similar torsional angles and accessibility within these matching secondary structure blocks are then selected for comparison.

Cliques indicate blocks within which residues in matching secondary structures should be aligned. Gaps between these blocks are also possible locations for the residue alignment algorithm to search. The rest of the score matrix can be ignored. This typically gives a significant reduction in the number of residue pairs that must be compared in the first pass of the DDP algorithm. As well as speeding up the alignment, it also reduces the amount of noise in the summary score matrix accumulated in the first pass, as fewer nonequivalent residue pairs are compared. Similarly, once a domain has been matched in the multidomain structure, the block associated with that domain need not be subsequently searched. These restrictions on the search space result in much faster comparisons without significantly affecting the ability to recognise equivalents.

*Adapting the CATHEDRAL protocol to favour global matches over local motif matches.* The accuracy of the secondary structure–matching algorithm improves with clique size because for larger cliques there are more equivalent geometric relationships identified. This is because a clique that has *N* nodes contains *N*(*N* − 1) / 2 edges. Matches identified using GT are therefore more secure when the clique is large, independent of the residue similarity score. Furthermore, because the scoring scheme for graph-matching breaks down for the very small folds (fewer than three secondary structures [25]), to maintain the integrity of CATHEDRAL's predictions, these very small proteins are excluded by the algorithm.

As CATHEDRAL iterates toward a solution, the CATH database is repeatedly scanned. However, some large folds contain structural motifs that match well to small folds. These motif matches sometimes rank higher in the match list because the geometry is very well conserved, and the selection of these matches over equivalent folds can therefore confuse the identification of domain boundaries. This effect can be avoided by attempting to match only large domains first; that is, two passes of CATHEDRAL are performed. The first pass only allows matches to folds in CATH that have graphs of five or more nodes. Once CATHEDRAL has reached its termination, it is applied again to the folds in CATH that have graphs with three or four nodes.

This strategy results in the smallest folds only being compared against regions of the multidomain protein that are not part of a large fold, as well as typically increasing CATHEDRAL's speed by 50% or more since fewer searches are required. Hence, CATHEDRAL essentially assigns all large domains first before attempting to align smaller domains to any remaining unassigned regions. To aid the assignment of discontiguous domains, in the first iteration, the top hit is also required to be contiguous (i.e., the assigned region comprises one continuous sequence segment).

*Scoring the structural similarity of the domain region aligned by DDP.* To assess whether a given structural hit represents a true fold match within the multidomain protein, several measures of similarity are calculated. The structural similarity score returned by the DDP algorithm is normalised to lie in the range of 0–100 (with 100 for identical structures) irrespective of the protein sizes [44]. This score is based on similarities in the vectors between C_β_ atoms of equivalent residues in the aligned proteins and is normalised to take account of the size of the largest domain being compared.

A rigid body superposition of the structures is also generated from the equivalent residues identified by the alignment. RMSD of the aligned C_α_ atoms is calculated, and a cutoff can be imposed on the local structural similarity (see above) to select only the most similar residue pairs when generating the superposition of the structures. A cutoff of 30 (with 100 representing identical residues) is used to ensure the most equivalent residues pairs are used to calculate the SAS.

*Using an SVM to validate structural matches.* Determining domain boundaries in protein chains through iterative fold assignment presents several challenges. For example, there is the problem of mis-assigning folds that simply match a large structural motif that does not correspond to a significant “global” match to the domain region. Discontiguous domains can also present problems for structural alignment algorithms. Several similarity measures can be considered when gauging whether a match is valid. Manual experimentation can be used to explore and optimise the combination of these measures, or machine learning methodologies can be used. In CATHEDRAL, we exploited an SVM to perform the optimisation automatically and to determine when a significant domain structure match to a classified fold in CATH was occurring.

In addition to the similarity measures provided by the GT and DDP algorithms, we also considered other features (e.g., the proportion of residues matched between the two structures, and similarity in domain sizes) to help improve recognition of global similarity between domain structures. We used the SVMLight package [45] to combine these features using a linear kernel.

To train the SVM, 5-fold cross-validation was used to assess the performance of the SVM models. That is, the dataset was split into five sets, and each one was successively used as the test set, while the model was trained on the other four sets. This reduces any potential bias caused by random fluctuations in the composition of the training and test sets. The error cost for positive examples was weighted according to their ratio to negative examples.

Features included the raw score, E-value, and clique size (number of matched secondary structures) returned by the GT comparison. In addition, the raw score derived from the DDP algorithm together with the residue overlap (percentage of residues in the CATH domain aligned against the putative domain region), CATH domain size, sequence identity, and SAS. To improve the ability of the classifier to avoid bias toward one feature, each was normalised between 0 and 1.

*Identifying domain boundaries and handling discontinuous domains.* The individual DDP similarity score of equivalent residue pairs, normalised to lie between 0–100, indicates where residue similarity is good (high), where it is poor (low), and where it is nonexistent (residue score is zero). Since only individual domains from CATH are scanned against the multidomain structure, the alignment can be used to find domain boundaries, because the residue pair score falls to zero at the boundary.

When CATHEDRAL determines which fold to assign to a region of the protein chain, it is also making a judgment of where the domain boundaries lie. The fidelity of this latter process is arguably dependent on the structural similarity between the domain region in the chain and the domain it has matched in the library. Although domain boundaries can be assigned to the chain in the same step as taking the highest scoring hits to each region of the chain, the accuracy can be improved by modifying the boundaries once all assignments have been made.

Subsequently, domain assignments that contain regions of the chain that overlap with one another are processed as a last step in the protocol. Conflicts are resolved by assuming that the highest-scoring domain is most likely to have the correct boundaries. The boundaries of the overlapping domain are cropped to exclude the shared region. Second, some chains may contain small regions at the start and end that are unassigned. This is often fewer than 20 residues and is unlikely to contain another domain, or comprise an additional segment of a discontiguous domain. In these instances, CATHEDRAL assigns the extra residues at the beginning and end of the chain to the first and last domains, respectively. Similarly, some chains contain small regions between assigned segments. In these cases, CATHEDRAL splits the unassigned residues equally between the two neighbouring segments.

## Supporting Information

Text S1 Description of CATH Domain Structure Database (48 KB PDF)

## Abbreviations

DDP: double-dynamic programming
GT: graph theory
HMM: hidden Markov model
PDB: Protein Data Bank
PDP: protein domain parser
PUU: protein unfolding unit
RMSD: root-mean–squared deviation
ROC: receiver operating characteristic
SAS: structural alignment score
SVM: support vector machine
