# Supplementary material for: CATHEDRAL: A Fast and Effective Algorithm to Predict Folds and Domain Boundaries from Multidomain Protein Structures
Source: PLoS Comput Biol. 2007 Nov 30;3(11):e232. doi: 10.1371/journal.pcbi.0030232 (PMC2098860; doi:10.1371/journal.pcbi.0030232)
Supplement: Text S1 — (48 KB PDF) [file pcbi.0030232.sd001.pdf]

Appendix I

Description of CATH Domain Structure Database

CATH (Class, Architecture, Topology, Homology, [1]) is a hierarchical classification of the 3D structures deposited in the Protein Data Bank (PDB, [2]). There are five major classification levels in the CATH hierarchy. The top C level separates domains into different Classes based on the type of secondary structure within the domain, principally mainly-α helices, mainly β-strands or a mixture of the two, α-β. The A level divides the classes into different Architectures, which describes the shape of a protein, given by the secondary structure orientations in 3D, regardless of their connectivity e.g. the protein resembles a barrel or a sandwich. The T level divides these architectures into their distinct Topologies (folds). The topology of a protein details the specific connectivity amongst the secondary structures. At the Homology level, folds are further separated into superfamilies that have a high structural and sequence or functional similarity, indicating evolution from a common ancestor. The S level divides the homologous structures into Sequence families. Members of the individual S families have ≥35% sequence identity to at least one other relative and have very similar structures and functions.

Description of Structure Comparison Algorithms Employed in CATHEDRAL

Graph theoretical Approach for Comparing Protein Structures

A graph is a mathematical description of a system, representing both the layout of a system and how the individual components interact. In graph theory terminology, the components of the system are called “nodes” and the interactions are termed “edges”. The fold of a protein is readily described as a graph, particularly because secondary structures can be abstracted as vectors [3]. The nodes in such a graph are associated with each secondary structure vector and are denoted as either helix or strand. The edges within the graph are labelled by the geometric relationships between each pair of secondary structures: more specifically, the distance of closest-approach, dot-product angle and dihedral angle.

The transformation of structures into graphs allows a determination of the amount of overlap in the geometrical descriptions of two proteins, by constructing a correspondence graph. A clique is the part of this graph in which every node has an edge connected it to all the other nodes. In

CATHEDRAL, a standard Bron-Kerbosch search algorithm is used to detect the largest clique in the correspondence graph, which corresponds to a matching structural motif between the two proteins. Further details of this methodology can be found in [4].

A simple scoring function is utilised to measure the similarity between two structures [4]. This is based on the sizes of the two comparison proteins, the size of the clique and the percentage of equivalent residues within the clique (residue overlap). Distributions of scores returned from database scans using this approach exhibit an extreme value distribution in the tail. This allows numerical analysis to calculate the frequency with which any particular score could be obtained by chance. The resulting E-value has been shown to provide a consistent statistical description of comparisons across fold space and has no obvious biases towards certain folds, architectures or classes.

Overview Of Double Dynamic Programming for structural alignment

Double dynamic programming was first employed in the SSAP program developed by Taylor and Orengo in 1989. It uses the popular Needleman and Wunsch global dynamic programming algorithm on two levels of matrices. A single upper level matrix is used to accumulate possible alignment paths from the lower level matrices, which compare the structural environments of putative equivalent residues pairs.

A structural environment is described by the set of vectors from a given residue to all other residues in the same structure. Vectors are calculated between Cβ atoms and then transformed to a common co-ordinate frame defined by the tetrahedral geometry of the Cα atom. Dynamic programming is then used to align the vector sets for a pair of residues in each protein and if the cumulative score is sufficiently high, the alignment path through the score matrix is added to the upper level summary matrix. The top 20 highest scoring pairs are selected from this matrix, which is then reset to 0. The top 20 pairs are then re-compared and these paths are added to summary matrix. Finally, dynamic programming is used to determine the best alignment path through the summary matrix, giving the final similarity score between the proteins.

Description of Other Structure Comparison Algorithms Used in Assessing the Performance of CATHEDRAL

CE [5] identifies matching octapeptide fragments between structures, which share similar local geometry. These are described as aligned fragment pairs (AFPs) and are concatenated in succession to extend the alignment, with gaps permitted provided their length does not exceed 30 residues — to maintain the speed of the algorithm. CE then seeks the alignment with the best RMSD using dynamic programming and this is returned as a Z-score.

DALI [6] also uses a small fragment approach to construct its alignments. Six residue peptides are compared using contact maps and potentially equivalent pairs identified by searching for similar patterns of distances between residues. The Monte Carlo optimisation method is employed to search for equivalent sets of similar hexapeptide pairs to be concatenated into an alignment. DALI uses many initial alignments and searches for the best one based on the RMSD. Output includes a raw score, summed over all aligned residue pairs and a normalised z-score.

STRUCTAL [7] identifies an initial alignment between the structures and uses this to superimpose the structures by rigid body transformation to obtain a minimal RMSD. Subsequently, an optimal alignment is obtained through dynamic programming. Initial alignments are obtained in various ways, for example by considering the sequence similarity of the proteins or torsional angle similarity. An iterative approach is employed whereby alignments are refined by dynamic programming and this is followed by further superposition until a local optimum is converged upon. STRUCTAL provides statistical measure of significance of the final alignment produced in the form of a p-value.

LSQMAN [8] also adopts an iterative approach based on rigid body superposition. The first residue of each secondary structure element in the two structures is optimally superposed to give an initial transformation. Subsequently, the method seeks long alignments, of at least 4 residues, in which matching residues are within 6Å separation. These alignments guide a new superposition and the process is repeated in an iterative fashion, with the distance threshold being increased for each iteration. LSQMAN outputs a Z-Score to give a statistical interpretation of the alignments significance.

References

1. Orengo CA, Michie AD, Jones S, Jones DT, Swindells MB, Thornton JM (1997) CATH--a hierarchic classification of protein domain structures. Structure 5: 1093-1108.

2. Berman HM, Westbrook J, Feng Z, Gilliland G, Bhat TN, Weissig H, Shindyalov IN, Bourne PE (2000) The Protein Data Bank. Nucleic Acids Res 28: 235-242.

3. Richards FM, Kundrot CE (1988) Identification of structural motifs from protein coordinate data: secondary structure and first-level supersecondary structure. Proteins 3: 71-84.

4. Harrison A, Pearl F, Sillitoe I, Slidel T, Mott R, Thornton J, Orengo C (2003) Recognizing the fold of a protein structure. Bioinformatics 19: 1748-1759.

5. Shindyalov IN, Bourne PE (1998) Protein structure alignment by incremental combinatorial extension (CE) of the optimal path. Protein Eng 11: 739-747.

6. Holm L, Sander C (1993) Protein structure comparison by alignment of distance matrices. J Mol Biol 233: 123-138.

7. Gerstein M, Levitt M (1998) Comprehensive assessment of automatic structural alignment against a manual standard, the scop classification of proteins. Protein Sci 7: 445-456.

8. Kleywegt GJ (1996) Use of non-crystallographic symmetry in protein structure refinement. Acta Crystallogr D Biol Crystallogr 52: 842-857.
